# Tuberculosis preventive treatment in a single medical center and evaluation of the results

**DOI:** 10.3892/etm.2014.1988

**Published:** 2014-09-22

**Authors:** BEYHAN ÇAKAR, NALAN DEMIR, DEMET KARNAK, ŞEREF ÖZKARA

**Affiliations:** 1 Ankara Tuberculosis Control Dispensary No. 7, Ankara University School of Medicine, Ankara 06100, Turkey; 2 Department of Chest Diseases, Ankara University School of Medicine, Ankara 06100, Turkey; 3 Department of Chest Diseases, Atatürk Chest Diseases and Chest Surgery Educational and Training Hospital, Ankara 06280, Turkey

**Keywords:** tuberculosis, tuberculin skin test, isoniazid, prophylaxis, BCG scar, preventive treatment

## Abstract

The aim of the present study was to evaluate the application of tuberculosis preventive treatment (TB-PT). Demographic data, indications and results for cases that received TB-PT at the Ankara Tuberculosis Control Dispensary No. 7 between 2008 and 2011 were retrospectively evaluated. The ‘Prevention with Drugs’ registry at the dispensary was used. A total of 463 cases received TB-PT, with the indications including close contact with an active TB case (44%), positive tuberculin skin test (TST) in a child <15 years-old (25%) and immunosuppressive therapy (31%). The immunosuppressed group (n=144) were administered steroids (10%) or tumor necrosis factor (TNF)-α inhibitors (90%). Indications of TST conversion and sequela lesions were not observed among the cases. The male/female ratio was 106/98 for cases with TB close contact, 61/54 for TST-positive cases and 85/59 for immunosuppressed cases. The mean ages of these groups were 9±5.7, 9.5±3.8 and 38±14.9 years, respectively. TB-PT was completed in 364 cases (78.6%), and the rate of discontinuation due to adverse effects was 1% for TB close contact and 2% for TST-positive cases, but 5% for immunosuppressed cases. While the percentage of TB close contact cases receiving TB-PT decreased during the four-year study period, the percentage of cases with immunosuppression (in particular patients using TNF-α inhibitors) increased. Among the studied cases, only two subjects developed active TB. The first case involved a 1.5-year-old female that had close contact exposure to TB from a parent, while the other case involved a 14-year-old TST-positive male (induration size,16 mm). In conclusion, patients receiving TB-PT should be monitored and/or followed-up carefully to control any side-effects from the treatment and development of active TB.

## Introduction

Tuberculosis (TB) is a major public health issue in Turkey, and worldwide. Preventive treatment (TB-PT) is essential in the fight against TB, and the determination and treatment of index cases. TB-PT aims to prevent the occurrence of active TB in patients with latent TB infection (1). Prior to TB-PT, the tuberculosis skin test (TST), also known as the purified protein derivative (PPD) test, is used to determine whether an individual has developed an immune response to the bacterium that causes tuberculosis. The TST is based on the fact that infection with *Mycobacterium tuberculosis* bacterium causes a delayed-type hypersensitivity skin reaction to certain components of the bacterial reaction with the skin. PPD is initiated when T cells, which have been sensitized by prior infection, are recruited to the skin site. Here, they release lymphokines which induce induration (a hard, raised area with clearly defined margins at and around the injection site) through local vasodilation leading to edema, fibrin deposition and the recruitment of other types of inflammatory cells to the area. TB-PT has been part of the TB control program in Turkey for decades. Since 2003, with the publication of the new TB Guidelines in Turkey, contact tracing and the use TB-PT has increased (2). TB cases that develop from contact with other active TB cases have decreased following this practice.

The Turkish national guidelines (3) on the administration of preventative treatment identify the following groups at a high risk for TB infection: Children <15 years-old showing a positive response to the TST; patients with TST conversion, patients <35 years-old who have come into close contact with active pulmonary TB cases; and patients with other conditions that are vulnerable to developing active TB, including HIV, diabetes mellitus, scalp, neck, blood and lymph system cancers, low weight, silicosis or apical fibrotic lesions, patients using tumor necrosis factor (TNF)-α inhibitors or corticosteroids, and patients that have undergone mastectomy, jejunoileal bypass and organ transplantations (2,3).

The aim of the present study was to evaluate the application of TB-PT in a single medical center between 2008 and 2011.

## Materials and methods

### Methodology

Demographic data, indications for treatment, BCG vaccine scarring, TST values and therapy results of patients who received TB-PT between 2008 and 2011 at the Ankara Tuberculosis Control Dispensary No. 7 (Ankara, Turkey) were evaluated retrospectively. The ‘Prevention with Drugs’ registry in the dispensary was used. Prior to the initiation of TB-PT, 0.1 ml 5 tuberculin units (0.1 ml) of tuberculin were intradermally injected on the inner forearm of the patients, in accordance with the Mantoux technique. The induration size was measured after 72 h and assessed according to the Turkish national guidelines (3). For BCG-vaccinated patients, an induration size of ≤5 mm was defined as negative, 6–14 mm was considered to be associated with the vaccine or suspicious, and an induration of >15 mm was considered positive. For non-vaccinated subjects, an induration of 6–9 mm was regarded as suspicious, while >10 mm was considered positive. A chest X-ray was performed in all cases prior to treatment, and TB-PT was administered to all cases without active TB disease (3). This retrospective study was approved by the Department of Tuberculosis Control at the Ministry of Health (no. 26475; Ankara, Turkey), and written informed consent was provided by the patients.

## Results

### Patients

A TST was performed in 5,855 cases and TB-PT was administered to 463 cases between 2008 and 2011. The indications for TB-PT included close contact with an active TB case (44%), a positive TST in a child <15 years-old (25%) and the administration of immunosuppressants (31%). Table I demonstrates the yearly indications for TB-PT within the 2008–2011 period. Immunosupression in 144 patients was caused by the use of steroids (10%) and TNF-α inhibitors (90%), administered to treat conditions, such as rheumatoid arthritis, ankylosing spondylitis, psoriasis, Behçet’s disease and Crohn’s disease. TNF-α inhibitors were most commonly used to treat rheumatoid arthritis (52%). TB-PT was not administered to cases with TST conversion and sequela lesions. The male/female patient ratios were 106/98 for cases of close contact with active TB, 61/54 for TST-positive cases and 85/59 for immunosuppressed cases. The mean age was 9±5.7 years (age range, 1–35 years) for cases with close contact with active TB, 9.5±3.8 years (age range, 1–15 years) for TST-positive cases and 38±14.9 years (age range, 1–77 years) for immunosuppression cases. The gender and age distributions of TB-PT cases are reported in Table II.

### Preventive therapy

The completion rate of TB-PT was 78.6% (n=364), with 65 patients not completing the therapy (14.0%) and 13 cases transferred to a different center. The results of TB-PT are summarized in Table III.

### TB development

**Figure 1:**
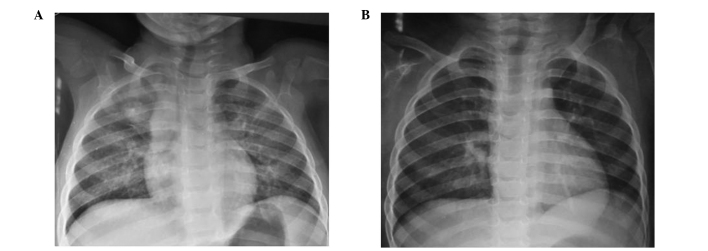
Chest X-rays of a 1.5-year-old female with a tuberculin skin test induration of 10 mm (BCG-vaccinated), (A) during diagnosis of pulmonary tuberculosis (TB) and (B) following treatment for pulmonary TB disease. The father was identified as a smear-positive case of pulmonary TB, and the child was diagnosed with active pulmonary TB after 120 days of TB-PT. Bilateral infiltrations, a pulmonary nodule at the right upper zone and a fracture of the clavicula were identified on the chest X-ray.

**Figure 2:**
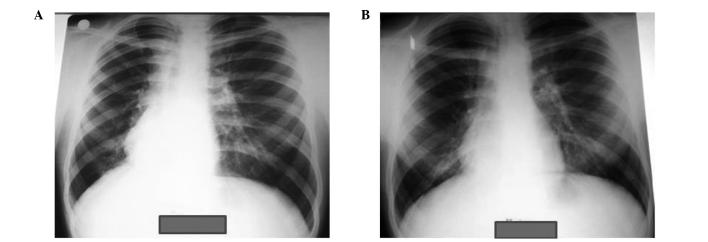
Chest X-rays of a 14-year-old male with a positive tuberculin skin test induration of 16 mm (BCG-vaccinated) and dextrocardia, (A) prior to and (B) following preventative treatment. Pneumonic infiltration was detected at the follow-up in the right lower zone of the chest X-ray after 50 days of isoniazid treatment.

Active TB developed in two cases during the course of the TB-PT, despite showing normal chest X-rays prior to isoniazid (INH) therapy. The first case involved a BCG-vaccinated female aged 1.5 years, who exhibited a TST induration of 10 mm. The father was identified as a smear-positive case of pulmonary TB, and the child was diagnosed with active pulmonary TB after 120 days of TB-PT. The second case involved a 14-year-old TST-positive male (16 mm induration; BCG-vaccinated) with dextrocardia, who developed active pulmonary TB after 50 days of treatment. Chest X-rays of the two cases with developing active TB are presented in Figs. 1 and 2. Standardized active TB treatment for children (two months of INH, rifampin and pyrazinamide plus four to seven months of INH and rifampin) was administered to these cases and completed according to the national guidelines.

### Therapeutic outcomes

The rate of TB-PT discontinuation was 1% for patients with close contact to active TB, 2% for TST-positive patients receiving INH prophylaxis for six months, and 5% for immunosuppressed patients receiving INH prophylaxis for nine months. Between 2008 and 2011, the percentage of TB-PT indications in patients with TB close contact decreased, while TB-PT in immunosuppressed patients increased.

## Discussion

Alongside early diagnosis and treatment, BCG vaccination and TB-PT are crucial in the fight against TB. Since 2006, single-dose BCG vaccinations have been applied to the general population under the Vaccination Program of the Turkish Ministry of Health, as opposed to the two-dose vaccinations applied prior to 2006. Children receive BCG vaccinations within the first three months of their life, unless any contraindications exist. After this age, a BCG vaccination is only applied according to the TST reaction, while after six years of age, BCG vaccinations are not administered. Active TB should be excluded prior to TB-PT, and periodical liver function tests (LFTs) should be performed following the initiation of treatment.

TB-PT should be discontinued if LFTs reveal a five-fold elevation of the basal value or three-fold elevation of the upper limit of aspartate aminotransferase, symptoms of nausea and vomiting or elevated levels of bilirubin and transaminase, unless the elevation is associated with other causes, such as viral hepatitis (2). The Tuberculosis Control Society of the Turkish Ministry of Health recommends TB-PT with INH for six months at 10 mg/kg/day (maximum 300 mg/day). In cases of HIV-positive patients or immunosuppression, the duration of the TB-PT is nine months (2,3).

Latent tuberculosis infection (LTBI) can be detected with immune-based tests, including the TST and interferon-γ release assay. Therapy in TST-positive patients can reduce the subsequent risk of disease reactivation and development of active TB. The effectiveness of INH treatment for LTBI, as measured in randomized controlled trials, varied between 25 and 92% (4–8). The current standard therapy using INH reduces the risk of active TB by up to 90%, if administered daily for nine months. However, the long therapy duration is disapproved by patients, and the risk of serious adverse effects, including hepatotoxicity, discourages patients and clinicians. As a result, therapy completion is <50% in a number of programs, and the problems associated with INH have stimulated the development and evaluation of several shorter regimens. An alternative therapy is the daily administration of rifampin and pyrazinamide for two months; however, this regimen is not currently used due to unacceptably high rates of hepatotoxicity and poor tolerability. A combination of INH and rifampin, administered for three or four months, has demonstrated an efficacy equivalent to six months of INH therapy, albeit with somewhat increased hepatotoxicity. Four months of rifampin treatment has an efficacy that is at least equivalent to six months of INH therapy; however, there are inadequate trial data available on efficacy. The safety of a four-month regimen of rifampin has been demonstrated in numerous studies. Recently, a large-scale trial evaluated a three-month INH regimen with weekly administration of rifapentine, under direct observation. This regimen may be promising in the treatment of LTBI if found as effective as INH treatment; however, the study results are yet to be published (4–8).

Chronic viral hepatitis (CVH) was not established as a risk factor for INH hepatotoxicity during TB-PT of CVH patients (9). Dobler and Marks reviewed the clinical files of patients that received TB-PT between 2000 and 2010. Out of 216 patients who commenced INH treatment for TB-PT, 163 (75%) completed the six-month treatment. Of the patients who did not complete the treatment, 53% dropped out after three months of treatment (10). A prospective cohort study demonstrated that only 53% of patients that began LTBI treatment completed the therapy (11). Horsburgh *et al* performed a retrospective trial at 68 clinics providing LTBI treatment, and observed that less than half the patients beginning the nine month LTBI treatment completed the therapy (12). According to the retrospective analysis performed by Kwara *et al* at a TB clinic in the USA in 2003, of the 845 patients with LTBI, 690 patients (81.6%) initiated a nine-month INH therapy. Only 426 patients (61.7%) completed the therapy, and follow-up was not possible for 246 patients (35.6%). Treatment was discontinued in 18 patients (2.6%), and it was primarily younger patients that failed to complete the therapy (13). In an additional study, adults that were not infected with HIV began treatment for LTBI at two specialist TB units in Spain. Of the 599 individuals that initiated treatment, 484 patients (80.8%) completed the treatment course. In patients under <36 years-old, short treatment regimens were not associated with improved treatment completion rates, when compared with the six-nine-month INH therapy (14). A systematic review of two databases (MEDLINE and EMBASE), stratifying patients according to age, aimed to determine the age-related risk of INH and rifampin hepatotoxicity under the recommended LTBI treatment regimens. Hepatotoxicity rates were low; however, the rates were higher among patients aged ≥35 years (1.7%) when compared with patients aged <35 years (0.2%) (15).

A survey of 217 LTBI patients in the USA revealed that only 28.6% of the patients finished at least six months of INH therapy under usual clinical conditions (16). In addition, a previous study performed on 1–18 year-old Mexican immigrant children revealed that out of 150 children with LTBI, 111 individuals (74%) completed INH treatment, 13 (9%) were transferred to a different center, while four children (3%) did not begin the treatment. One of the patients receiving treatment developed INH hepatitis (17).

Furthermore, a previous study reviewed the medical records of 474 LTBI patients placed on a four-month rifampin or nine-month INH treatment course between 2000 and 2003. The rifampin treatment was completed by 80.5% of the patients, while the INH treatment was completed by 53.1%. Patients receiving rifampin exhibited fewer reactions to the treatment and were significantly more likely to complete the therapy when compared with the patients receiving INH (18).

The present study revealed that the overall TB-PT therapy completion rate was 78.6%. More specifically, the completion rate was 77% for patients with close contact to active TB, 89% for TST-positive patients and 72% for immunosuppressed patients. The completion of long-term LTBI treatment was lowest in patients with immunosuppression. The rates of TB-PT discontinuation due to adverse effects were 1% for patients with TB close contact, 2% for TST-positive patients and 5% for patients with immunosuppression. The percentage of cases with TB close contact undergoing treatment decreased, while the immunosuppression cases (particularly using TNF-α inhibitors) increased in the four-year study period. The results of the present study, with regard to the rate of chemoprophylaxis completion, are comparable with those of previously published studies (10–13).

In conclusion, patients receiving TB-PT should be monitored and/or followed-up carefully to control any side-effects from the treatment and the development of active TB.

## Figures and Tables

**Table I:** Number and percentage of TB-PT indications in study subjects between 2008 and 2011.

	Year				
		
Indication	2008	2009	2010	2011	Total
TB close contact, n (%)	59 (53)	86 (56)	30 (28)	29 (31)	204 (44)
TST-positive, n (%)	37 (34)	34 (22)	24 (23)	20 (22)	115 (25)
Immunosuppression, n (%)	14 (13)	34 (22)	52 (49)	44 (47)	144 (31)
Total, n	110	154	106	93	463

TB, tuberculosis; TB-PT, tuberculosis preventive treatment; TST, tuberculin skin test.

**Table II:** Subject characteristics according to the indications of TB-PT.

Parameter	TB close contact	TST-positive	Immunosuppression
Female, n (%)	98 (48)	54 (47)	59 (41)
Male, n (%)	106 (52)	61 (53)	85 (59)
Mean age ± SD, years	9.0±5.7	9.5±3.8	38.0±14.9
Age range, years	1–35	1–15	1–77

TB, tuberculosis; TB-PT, tuberculosis preventive treatment; TST, tuberculin skin test; SD, standard deviation.

**Table III:** Results of TB-PT according to indications (number and percentage).

Parameter	Therapy completion	Default	TB diagnosis	Mortality	Center transfer	Other* [adverse effects]**	Total
TB close contact, n (%)	158 (77)	38 (19)	1 (1)	0 (0)	3 (1)	4 [1]**	204 (100)
TST-positive, n (%)	102 (89)	10 (8)	1 (1)	0 (0)	0 (0)	2 [2]**	115 (100)
Immunosuppression, n (%)	104 (72)	17 (12)	0 (0)	1 (1)	10 (7)	12 [5]**	144 (100)
Total, n (%)	364 (78.6)	65 (14.0)	2 (0.4)	1 (0.2)	13 (2.8)	18 (3.9)* [2.3]**	463 (100)

TB, tuberculosis; TB-PT, tuberculosis preventative treatment; TST, tuberculin skin test.

* Drug discontinuation rate due to adverse effects, doctor’s advise or lack of positive response to treatment.

** Drug discontinuation rate due to adverse effects only.
